# Supplementary figures and images for: Retrospective and statistical analysis of hand and forearm injuries in the Silesian pediatric population – study of post-traumatic X-rays in 2022
Source: PLoS One. 2025 Feb 14;20(2):e0318861. doi: 10.1371/journal.pone.0318861 (PMC11828379; doi:10.1371/journal.pone.0318861)

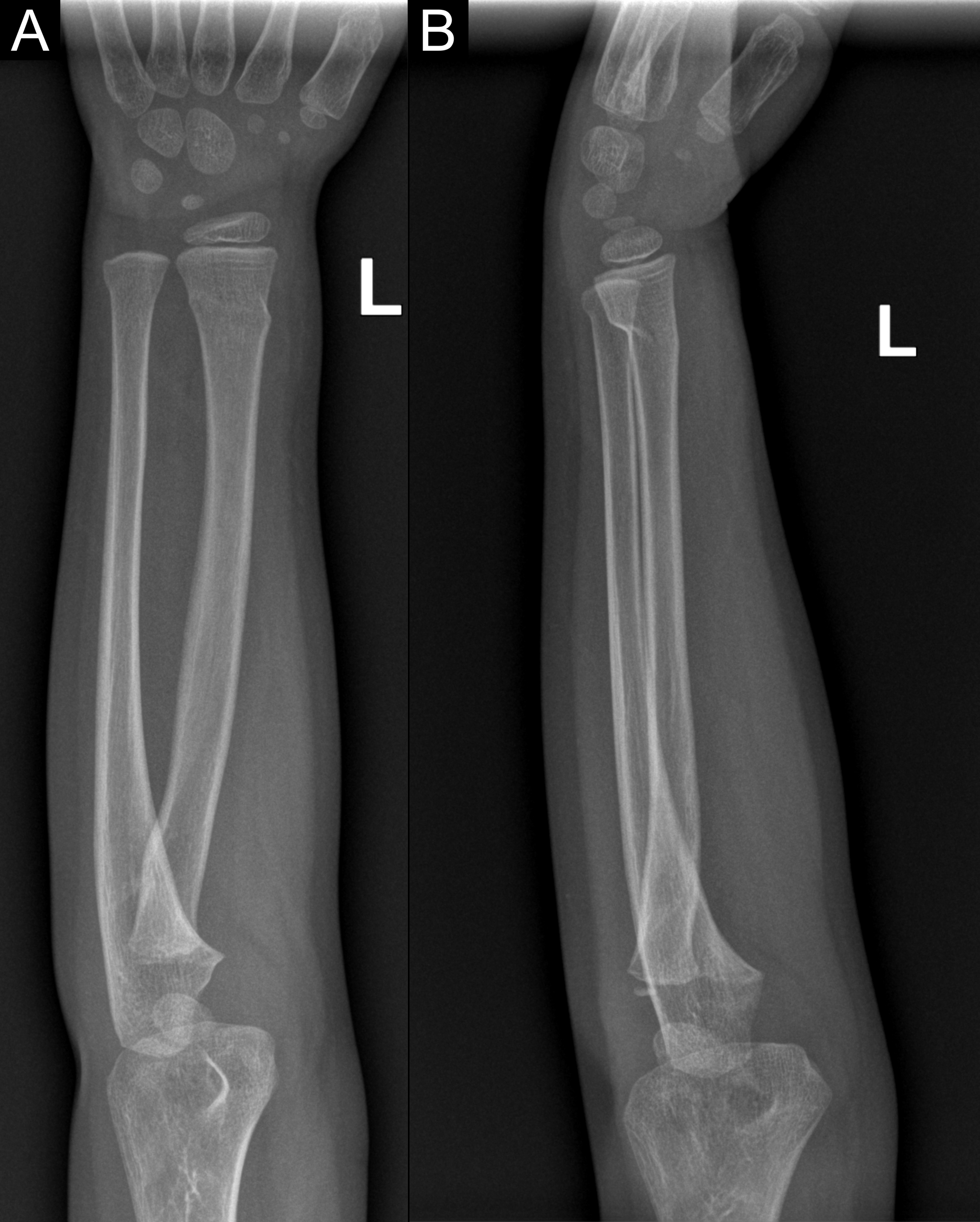

Supplement: S1 Fig — Torus-type fracture characteristic for children in the left distal radial metaphysis. (TIF) [file pone.0318861.s001.tif]

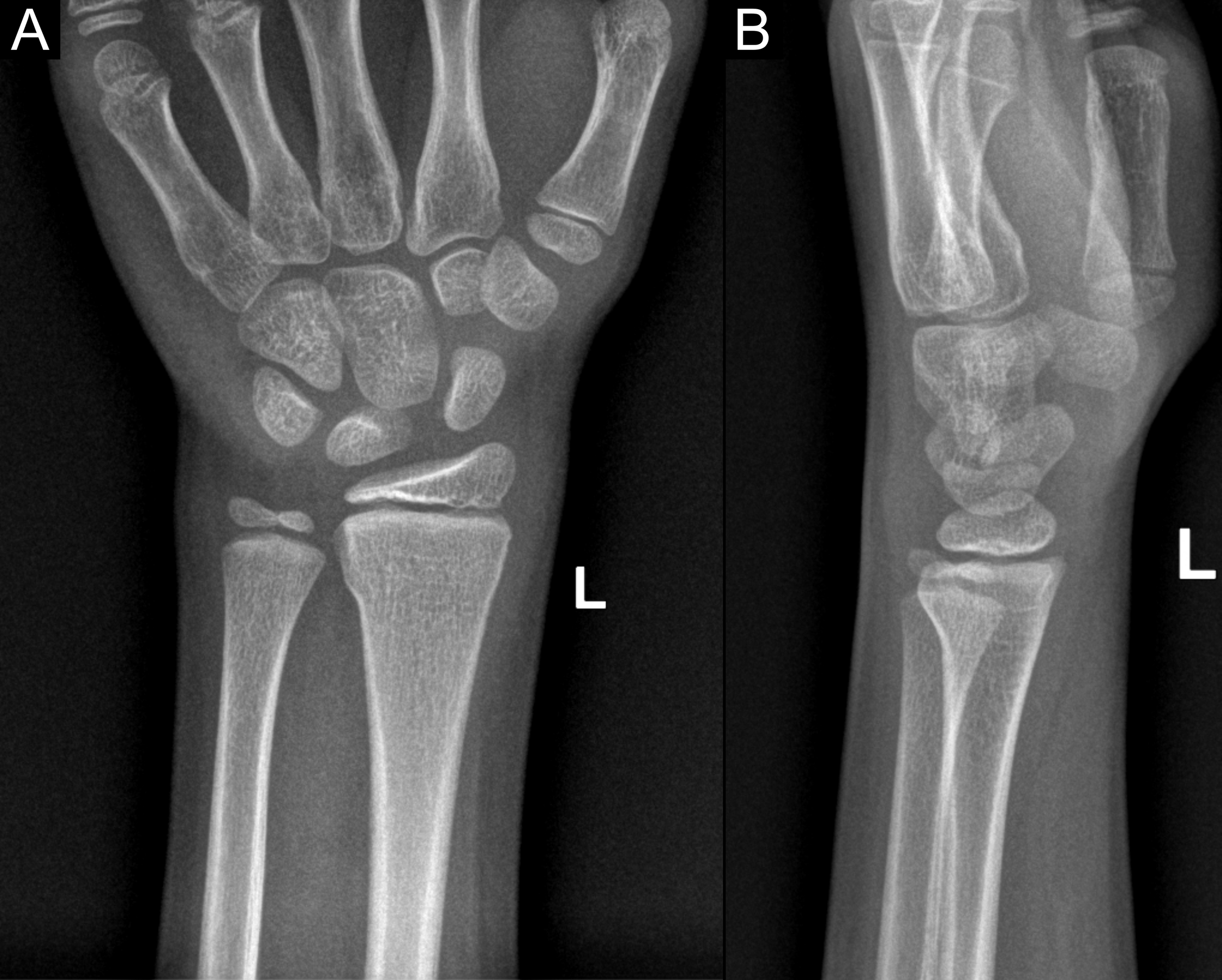

Supplement: S2 Fig — Angulation of the cortical layer in the distal metaphysis of the left radius. (TIF) [file pone.0318861.s002.tif]

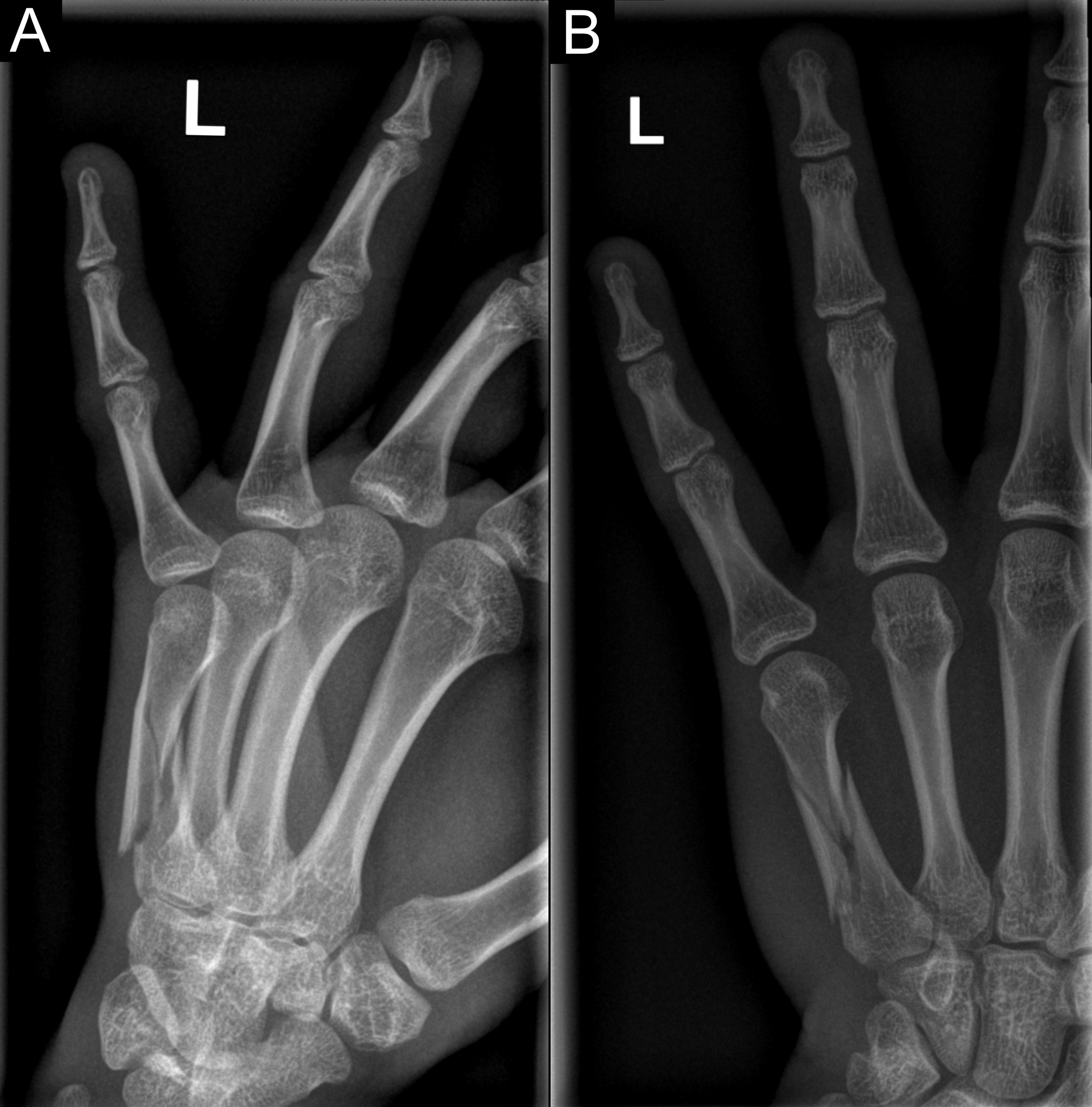

Supplement: S3 Fig — Multidirectional fracture of the shaft of the fifth metacarpal bone of the left hand with displacement of the fragment to the dorsal site. (TIF) [file pone.0318861.s003.tif]

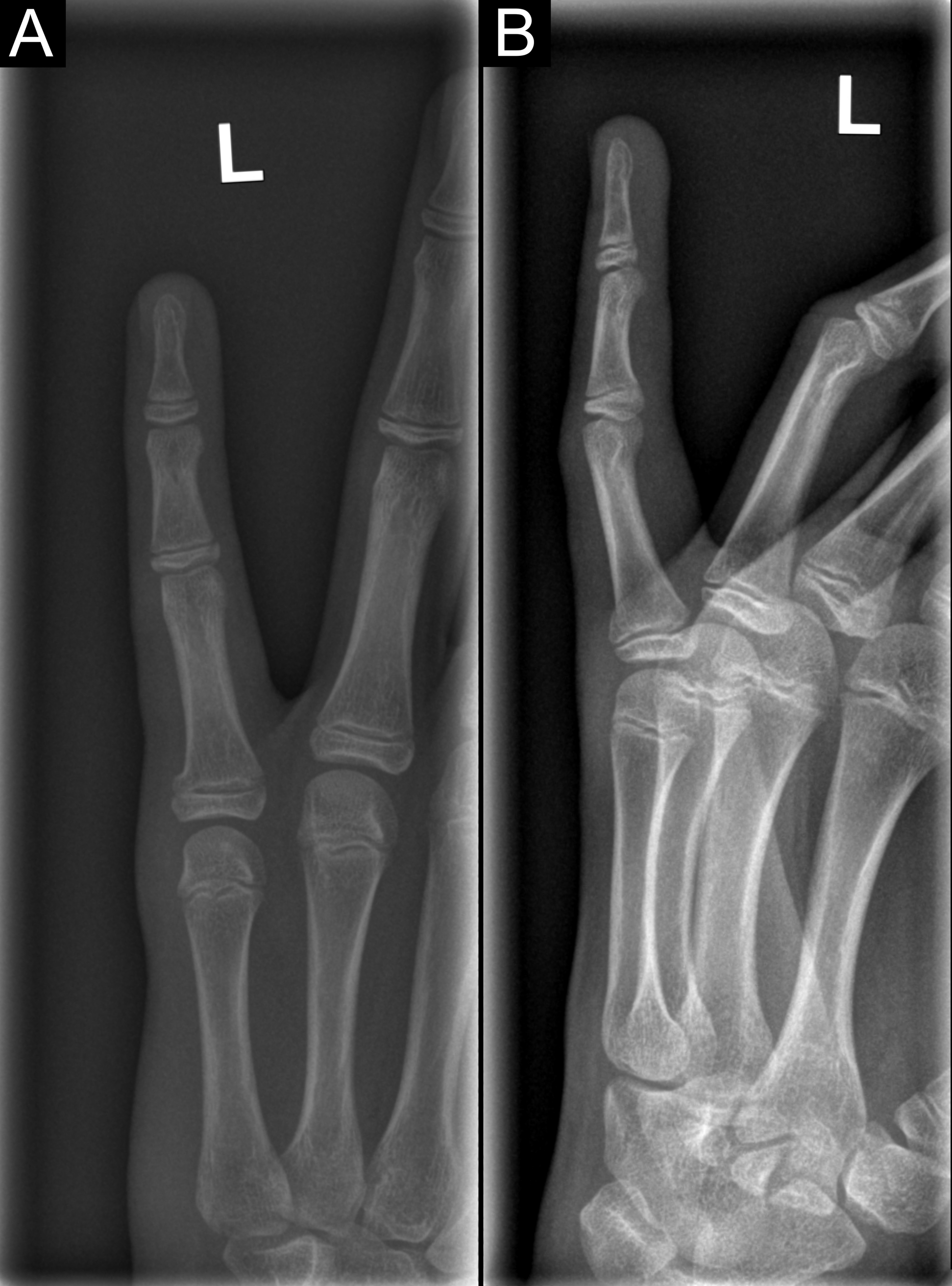

Supplement: S4 Fig — The injury under the angulation of the cortical layer at the base of the proximal phalanx of the fifth finger of the left hand. Visible ossification nuclei and growth cartilage. (TIF) [file pone.0318861.s004.tif]

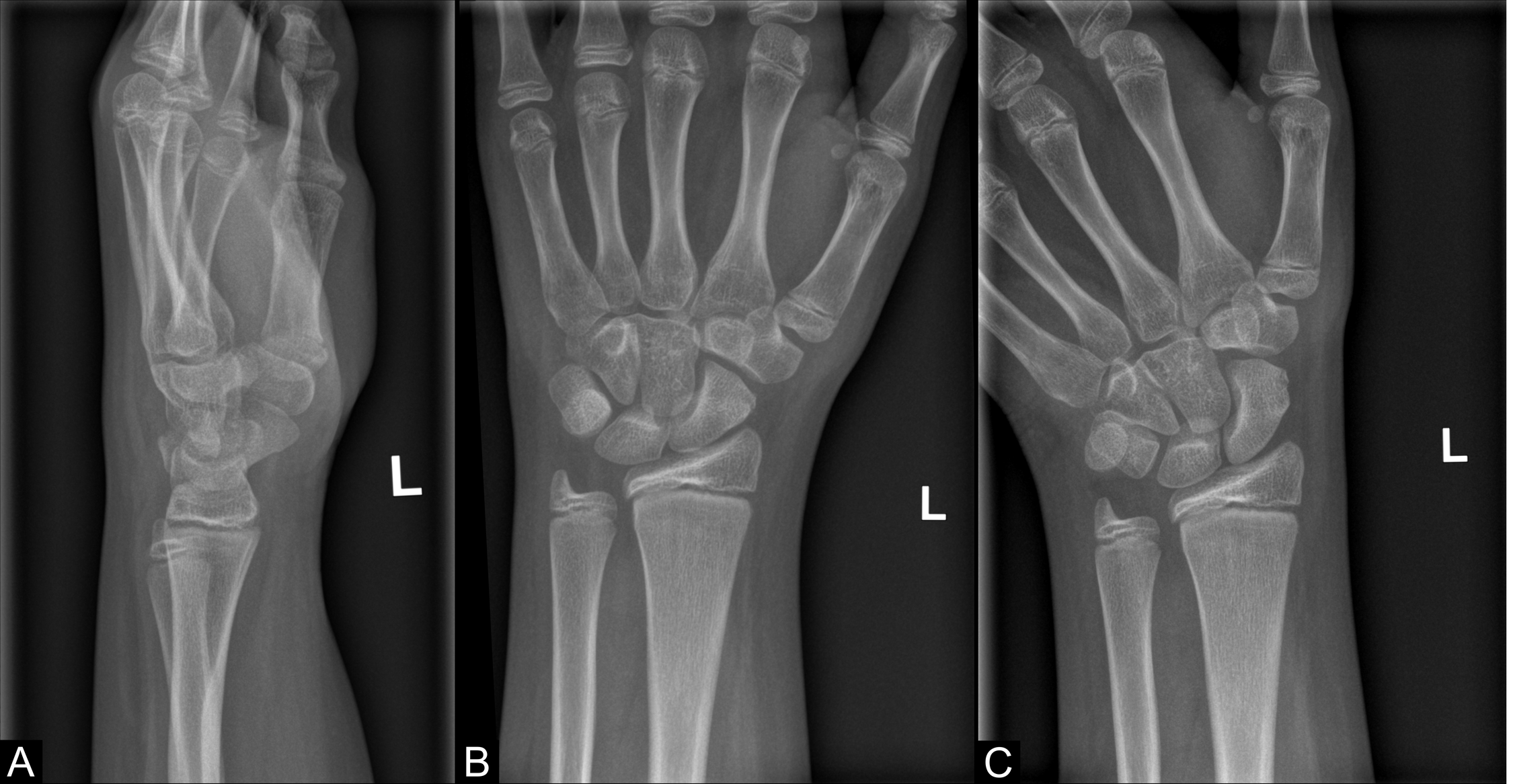

Supplement: S5 Fig — The injury can be seen only in the form of unevenness of the cortical layer in the waist of the bone. (TIF) [file pone.0318861.s005.tif]

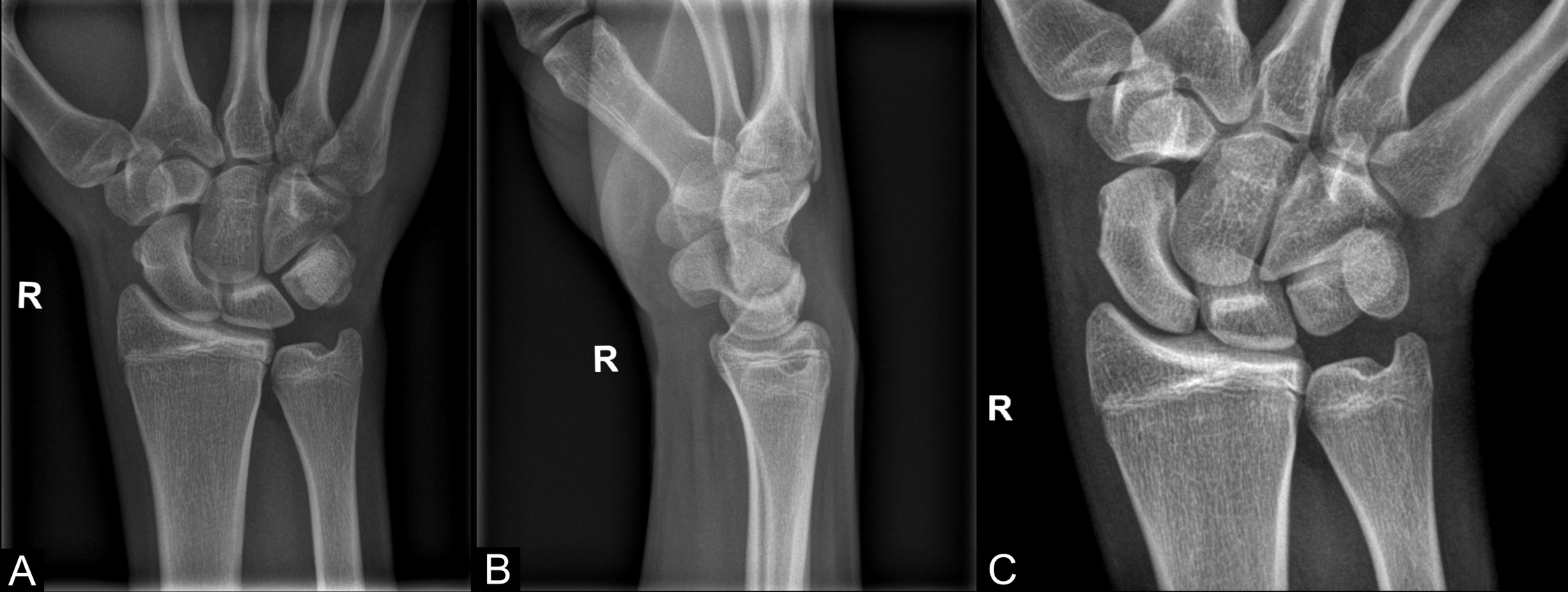

Supplement: S6 Fig — (TIF) [file pone.0318861.s006.tif]

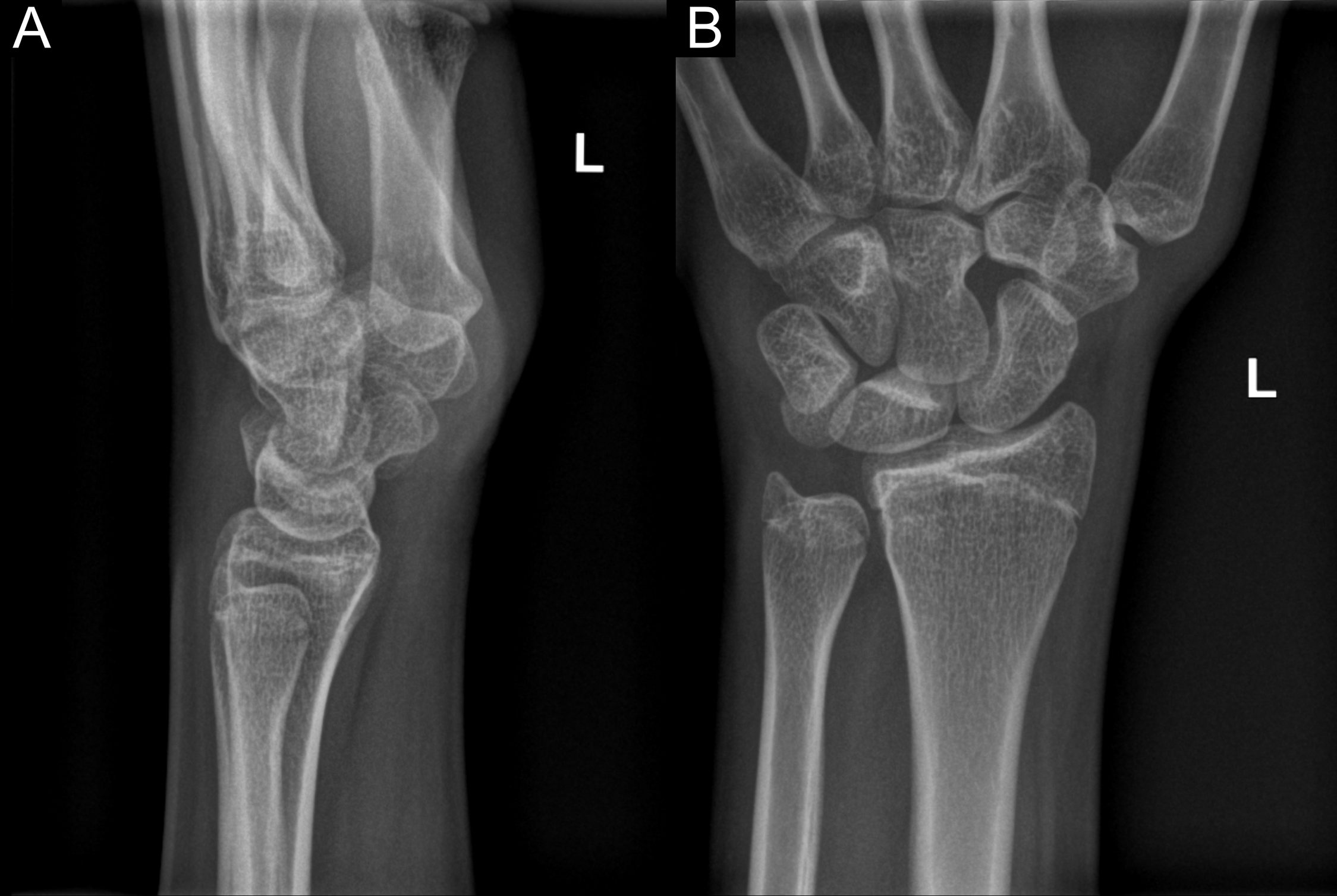

Supplement: S7 Fig — (TIF) [file pone.0318861.s007.tif]
