# Supplementary material for: Retrospective and statistical analysis of hand and forearm injuries in the Silesian pediatric population – study of post-traumatic X-rays in 2022
Source: PLoS One. 2025 Feb 14;20(2):e0318861. doi: 10.1371/journal.pone.0318861 (PMC11828379; doi:10.1371/journal.pone.0318861)
Supplement: Attachment — Submitted filename: Response to reviewers.docx [file pone.0318861.s008.docx]

Response to reviewers

Reviewer #1: The aim of this work is to conduct a comprehensive retrospective and statistical analysis of hand and distal forearm injuries in Silesian children. I think there are a few points that need to be revised.

1. When analysing the experimental data, could the authors add a comparison of the ethnicity of all the children who were treated in Silesia? If the experimental data obtained are all from the same ethnicity, the authors should mention it in the conclusion.

In the revised version, we included data containing information about ethnicity. However, we did not conduct comparisons or statistical tests due to the small number of individuals with non-Polish ethnicity and to keep our work more clear and concise.

2. Since the conclusions are based on statistical analysis, the contribution of this study is insufficient. To address this issue, could the authors provide more specific and practical conclusions, such as recommendations for prevention and treatment?

In revised version we included new conclusions subchapter.

3. The first sentence in Results Section “The study group consisted of 327 girls and 511 boys who were 11.4 ± 3.6 and 12.0 ± 3.8 years old, respectively (min. 0, max. 18 years).” is ambiguous. How can the minimum age in 12.0 ± 3.8 years old be 0?

In the revised version, we improved the clarity of the sentence, and it now includes information about the mean age.

4. In Table 1, it is better to add percentage statistics to the numbers of different fractures, for example, the number of fractures in forearm is 98 (xx%).

5. The author should provide detailed descriptions of the tables and figures in the paper to explain what they represent. In addition, authors should place these tables and figures in appropriate locations to make them easier for readers to read.

We reviewed our article layout and corrected mistakes according to commentary.

Reviewer #2: This is a meaningful study, which is helpful for the diagnosis and surgical treatment of fractures. However, I have some questions.

1. Patients were categorized into five age groups: 0-4 years, 5-8 years, 9-12 years, 12-16 years, and 17-18 years. What is the theoretical basis for the authors to divide the age into these groups? Are there any references?

In the revised version, we included a reference for the age group division.

2. The population included by the authors is children, and the authors should add data on the epidemiological characteristics of epiphyseal fractures.

The data included by the authors include hand injuries or fractures. The authors should clarify the difference between the two in the method.

In revised version we included clear definition for injury and fracture.

3. Does the data included by the authors include elbow dislocations in children?

In revised version we included a sentence in methodology regarding this information (Our study data doesn’t include elbow dislocations).

4. In the data included by the authors, a patient may have multiple fracture sites. The number of fracture sites will be more than the number of fracture sites. Therefore, the authors should clarify the epidemiological characteristics of patients with 2, 3 or even more fractures.

In the revised version, we included a sentence presenting data on the epidemiology of the multiple fracture group in our study.
