# Supplementary material for: Retrospective and statistical analysis of hand and forearm injuries in the Silesian pediatric population – study of post-traumatic X-rays in 2022
Source: PLoS One. 2025 Feb 14;20(2):e0318861. doi: 10.1371/journal.pone.0318861 (PMC11828379; doi:10.1371/journal.pone.0318861)
Supplement: Attachment — Submitted filename: Response-to-reviewers-091224.docx [file pone.0318861.s009.docx]

Response to reviewers

**Reviewer #1: Authors have addressed most of my concerns; however, I have one additional suggestion. Better to briefly list the contributions of this work at the end of the introduction section and to summarize them again in the conclusion section.**

Response to Reviewer Comments

We included a shortened list of this work's contributions at the end of the introduction section as suggested.

**Reviewer #2: Thank you for your reply. But I have some other questions.**

Response to Reviewer Comments

1. You mentioned that your research data does not include elbow dislocation. However, joint dislocation often occurs in children.

Thank you for raising this point. Unfortunately, the scope of the current study does not include data on elbow dislocation in children. The research was designed to focus on distal forearm wrist, and hand fractures, and joint dislocations were not within the predefined inclusion criteria.

2. Upper limb fractures in children are often accompanied by fractures of the lower limbs or even the spine. Do you collect such data?

We appreciate the suggestion to consider associated fractures in other anatomical regions. However, our dataset was limited to fractures of the upper limb. No systematic data on co-occurring fractures of the lower limbs or the spine were collected.

3. Did the author count the number of open and closed fractures in children?

The study does not include a detailed classification of fractures as open or closed. While such categorization could enrich the dataset and provide deeper insights into the severity and management implications, it was not part of the current research methodology.

4. Did the author count the complications of upper limb fractures in children? For example, are there any children with supracondylar humeral fractures combined with compartment syndrome?

Complications such as compartment syndrome in children with supracondylar humeral fractures were not analysed in the present study. While we recognize the clinical importance of evaluating such complications, they were outside the scope of this research. Future studies could incorporate this analysis to enhance the understanding of outcomes and complications associated with paediatric upper limb fractures.
